# Forward-Secure Linearly Homomorphic Signature Scheme in the Standard Model and Its Application

**DOI:** 10.3390/e28060706

**Published:** 2026-06-18

**Authors:** Linlin Wang, Zuling Chang

**Affiliations:** 1 School of Mathematics and Statistics, Zhengzhou University, Zhengzhou 450046, China; zuling_chang@zzu.edu.cn; 2 Institute of Mathematics, Henan Academy of Sciences, Zhengzhou 450046, China

**Keywords:** lattice, forward-secure, linearly homomorphic signatures, the minimal cover, key exposure

## Abstract

Linearly homomorphic signatures (LHSs) are widely used in scenarios such as network coding and the Internet of Things, but their security faces the serious threat of key leakage. To address this issue, this paper introduces a forward secure mechanism into LHSs, aiming to construct a linearly homomorphic signature (LHS) scheme that can resist the risk of key leakage. By combining the binary tree minimal cover set mechanism with lattice-based extension algorithms, we construct an LHS scheme that supports time-period key updates. We prove its forward secure unforgeability under the standard model (SM) by reducing it to the Short Integer Solution (SIS) problem. To the best of our knowledge, this scheme is the first provably secure lattice-based forward secure linearly homomorphic signature (FSLHS) scheme in the SM, filling a theoretical gap in existing research. Furthermore, we apply this scheme to a smart grid data acquisition system and verify its practicality through concrete performance analysis.

## 1. Introduction

With the widespread application of network coding and cloud computing technologies, integrity authentication during data transmission and processing has become one of the core challenges in cybersecurity. In network coding architectures [1,2], the combination of data packets by intermediate nodes is vulnerable to pollution attacks, threatening the reliability of the entire network. In cloud computing outsourcing scenarios [3], users need to ensure that any combination of outsourced data by the cloud server is verifiable and authentic. As a cryptographic primitive that allows linear operations on signed data while maintaining validation effectiveness, LHSs offer an excellent solution to these challenges. They enable intermediate nodes or cloud servers to perform secure linear combination directly on signed data vectors, while recipients or users can still verify the integrity and source authenticity of the final results. This approach ensures end-to-end security while enhancing network efficiency and computational scalability.

In 2002, Johnson et al. first provided a formal definition of homomorphic signatures and analyzed their security at the Cryptographers’ Track at the RSA Conference (CT-RSA). The related findings were systematically elaborated in [4]. In 2009, Boneh et al. [5] proposed the first LHS scheme suitable for network coding environments. In 2010, Gennaro et al. [2] further introduced an LHS scheme based on the RSA assumption. Subsequently, scholars conducted extensive research on the efficiency, security, and application scenarios of LHSs, proposing various schemes based on number-theoretic assumptions such as RSA and discrete logarithms [6,7,8,9]. With the advent of quantum algorithms such as Grover’s algorithm [10] and Shor’s algorithm [11], a growing number of researchers have shifted their focus to cryptography resistant to quantum computing attacks. Currently, lattice-based cryptography has become a key research focus among scholars due to its favorable properties and structural advantages.

In 2011, Boneh et al. [12] proposed the first lattice-based LHS scheme. By constructing signatures on a lattice modulo 2q, where the modulo 2 part encodes the message information and the modulo q part ensures security, this scheme cleverly resolves the difficulty of building homomorphic signatures over $F_{2}$. Its security is based on the $k-{\mathrm{SIS}}_{q,m}$ hard problem introduced in that paper. In the same year, Boneh and Freeman [13] used the intersection method to encode messages and functions into different lattice cosets, and employed the short basis delegation technique over ideal lattices to construct a polynomial homomorphic signature scheme based on ideal lattices. In 2013, Wang et al. [14] combined a preimage sampling algorithm with a family of hash functions to construct a new LHS scheme. Compared with the scheme in [12], this scheme significantly improved the public key size and signature size, while its security remained based on the standard SIS problem. In 2016, Chen et al. [15] ingeniously combined the Bonsai Tree technique with the intersection method of dual integer lattices to construct the first lattice-based LHS scheme that is provably secure in the SM, where the security relies on the SIS problem over lattices. In 2020, Lin et al. [16] designed two lattice-based LHS schemes using full-rank difference hash functions and linear homomorphic chameleon hash functions, improving the key and signature sizes over the scheme in [15]. In the same year, Cai et al. [17] abandoned the traditional trapdoor-based ’Hash-and-Sign’ paradigm and instead built their scheme based on the SIS hard problem, utilizing the Fiat-Shamir with Aborting framework and uniform sampling techniques. This scheme not only achieves side-channel resistance by avoiding Gaussian sampling but also significantly reduces the sizes of public keys and signatures. In 2024, Chen et al. [18], based on the scheme in [15] and combining pseudorandom function techniques with key homomorphic algorithms, designed the first lattice-based almost tightly secure LHS scheme, which only satisfies unforgeability under selective-tag static-message attacks. Subsequently, Gou et al. [19] introduced the NewBasis and Decompose algorithms to improve the signature size, efficiency, and security of this scheme, constructing a tightly secure LHS scheme, and proved that in addition to satisfying the security properties of [18], this scheme also achieves existential unforgeability under chosen-message attacks.

The security premise of LHSs relies on the confidentiality of keys. However, in dynamic environments such as network coding or cloud computing, the risk of key leakage is high, which could lead to the forgery of all historical data. Forward-secure mechanisms address this issue by periodically updating keys, ensuring that key leaks do not compromise past signatures and limiting the damage to the current period. This technology serves as a core enabler for achieving long-term secure network authentication systems.

The concept of forward-secure signatures was initially proposed by Anderson [20], and later formally defined and instantiated by Bellare and Miner [21], who constructed the first specific scheme. In 2000, Abdalla et al. [22] addresses the problem of excessive computational overhead in the key update process in [21] and proposes a new scheme based on a “certification tree” structure. This scheme uses the underlying signature algorithm to sign tree nodes to generate child node keys, thereby achieving key evolution over time. Furthermore, the security proof of this scheme relies on the unforgeability of the underlying signature algorithm, laying an important foundation for subsequent forward-secure scheme designs based on tree structures. In 2001, Itkis et al. [23] proposes the first forward-secure digital signature scheme with optimal efficiency in both signing and verification. Based on the Guillou–Quisquater signature mechanism, the scheme requires only two modular exponentiations to complete signing and verification, while keeping key and signature sizes compact, significantly outperforming previous forward-secure schemes. Subsequently, a series of improvements were made to enhance the efficiency of forward-secure signatures [24,25,26]. To address the key leakage issue in lattice-based signature systems, forward security mechanisms have been introduced, leading to the construction of a series of forward-secure signature schemes based on lattices. In 2014, Zhang et al. [27] combined the key update algorithm in Reference [28] with lattice-based delegation techniques to propose the first lattice-based forward-secure identity-based signature scheme. In 2019, Ling et al. [29] constructs the first lattice-based forward secure group signature scheme. This scheme utilizes the Bonsai tree structure to realize a scalable key evolution mechanism, and combines zero-knowledge proof techniques to prove full anonymity and forward-secure traceability under the SIS and LWE hard problem assumptions in the random oracle model. In 2020, inspired by [29], Le et al. [30] addresses the key leakage problem in blind signatures by using a binary tree data structure to organize time periods and employing the minimal cover set mechanism to manage key update paths. Combining the Fiat-Shamir transform and rejection sampling techniques, it constructs the first lattice-based forward-secure blind signature scheme. This scheme achieves forward security based on the SIS problem while preserving the blindness property of signatures. Inspired by [29,30], this paper applies the binary tree-based minimum covering set mechanism and lattice-based extension techniques to lattice-based LHSs, constructing the first FSLHS scheme in SM.

To the best of our knowledge, there are currently only two LHS schemes related to forward security. In 2024, Wu et al. [31] first introduced forward security into LHS and proposed an identity-based FSLHS scheme using fixed-dimension lattice delegation techniques and a family of additive homomorphic hash functions. In 2025, Wu et al. [32] utilized bilinear maps and the computational Diffie-Hellman (CDH) hardness assumption to design a FSLHS scheme through binary tree key evolution techniques. The core idea is to regularly update the private key and securely delete old keys to ensure the security of historical signatures. However, the security of both schemes is proven in the random oracle model (ROM), implying that their security may not be guaranteed in practical applications. To address the research gap of provably secure FSLHS schemes in the SM, this paper proposes a lattice-based FSLHS scheme and proves its forward security and unforgeability in the SM.

**Our contributions and techniques**: To mitigate the impact of key leakage in lattice-based LHS schemes, we incorporate a forward-secure mechanism and propose the first FSLHS scheme in the SM. Technically, we employ a complete binary tree to associate each node with a distinct time period. The root key is generated using the **TrapGen** algorithm, and keys for subsequent periods are derived via the **ExtBasis** algorithm. After each key update, the previous key is irrevocably deleted, thereby achieving forward security. We provide the formal definition and security model for FSLHS, and prove that the proposed scheme is forward-secure unforgeable in the SM under the hardness of the SIS problem.

**Article Structure**: The remaining sections of this paper are organized as follows: Section 2 introduces the basic knowledge and symbolic notation used in this paper. Section 3 presents the formal definition and security model of the FSLHS scheme. Section 4 elaborates on the specific algorithmic procedures of the proposed scheme and provides corresponding security proofs. Section 5 compares and analyzes the strengths and weaknesses of our scheme against existing signature schemes of the same type. Section 6 presents a concrete application scenario of the proposed signature scheme and analyzes the feasibility of the scheme in this scenario. Section 7 summarizes the work of this paper and discusses future research directions.

## 2. Preliminaries

In this section, we present the basic definitions, lemmas, algorithms, and the meanings of the symbols used in this paper.

### 2.1. Notation

This section mainly explains the symbols appearing in this paper and their meanings, as shown in Table 1.

### 2.2. Definitions and Lemmas

In lattice cryptography, an *m*-dimensional lattice Λ is a discrete additive subgroup within the *m*-dimensional real space $R^{m}$. Beyond this, the following class of lattices is frequently employed.

**Definition** **1** (*q*-ary lattices)**.** *Let $X\in Z_{q}^{n\times m}$, $y\in Z^{n}$. Two q-ary lattices are*

$$\begin{array}{c} \left(1\right)\;\Lambda_{q}^{⊥}\left(X\right)=\left\{u\in Z^{m}:X\cdot u\equiv 0\left(\mathrm{mod}q\right)\right\}\;and\;\left(2\right)\;\Lambda_{q}^{y}\left(X\right)=\left\{u\in Z^{m}:X\cdot u\equiv y\left(\mathrm{mod}q\right)\right\}. \end{array}$$

*For any $x\in Z^{m}$, $\Lambda_{q}^{y}\left(X\right)=\Lambda_{q}^{⊥}\left(X\right)+x$ such that X·x≡y(modq). Here, 0 is the zero vector.*

**Definition** **2** (SIS problem [33])**.** *Given an integer modulus q>2, an integer m=poly(n), a dimension n and a randomly generated matrix $A\overset{\$}{⟵}Z_{q}^{n\times m}$. Let β be a parameter such that 0<β<q. The goal is to find a vector $x\in Z^{m}\setminus \left\{0\right\}$ satisfying A·x≡0(modq) with ‖x‖≤β.*

**The hardness of SIS**: With the parameter setting where m=poly(n), β>0, and *q* is a prime satisfying $q\geq \beta \cdot \omega (\sqrt{n\log n})$, it was proven in [34] that breaking the average-case ${\mathrm{SIS}}_{q,m,\beta }$ is at least as hard as solving worst-case ${\mathrm{SIVP}}_{\gamma }$ (and related lattice problems) to within an approximation factor of $\gamma =\beta \cdot \tilde{O}\left(\sqrt{n}\right)$. Therefore, solving the SIS problem is hard, and the security of this scheme is based on this assumption. Where SIVP is the abbreviation for the Shortest Independent Vectors Problem.

**Definition** **3.** *Let $s\in R^{+}$ and $w\in R^{n}$. The Gaussian function is $\rho_{s,w}\left(x\right)=e^{-\frac{\pi }{s^{2}}{\left\|x-w\right\|}^{2}}$, where w is the central vector, and s is the Gaussian parameter. The Gaussian distribution measure $D_{\Lambda ,s,w}$ on the lattice* Λ *is expressed as $D_{\Lambda ,s,w}=\frac{\rho_{s,w}\left(x\right)}{\rho_{s,w}\left(\Lambda \right)}$, where $\rho_{s,w}\left(\Lambda \right)=\sum_{z\in \Lambda }\rho_{s,w}\left(z\right).$ and w can be omitted when w=0.*

In the proof of the correctness and security of the scheme in this paper, the following lemmas will be utilized.

**Lemma** **1** ([34])**.** *Let* Λ *be an m-dimensional lattice, and T be a basis of* Λ*. If $s>\;∥\tilde{T}∥\omega \left(\sqrt{\log m}\right)$, then for any center $v\in R^{m}$, we have:*

$$\mathrm{Pr}\left\{∥x-v∥>s\sqrt{m}:x\leftarrow D_{\Lambda ,s,v}\right\}\leq \mathrm{negl}\left(m\right).$$

*Equivalently, with overwhelming probability over $x\leftarrow D_{\Lambda ,s,v}$, it holds that $∥x-v∥\leq s\sqrt{m}$.*

In Ref. [12], Boneh and Freeman proved that a linear combination of several mutually independent vectors following discrete Gaussian distributions still follows a discrete Gaussian distribution. The detailed conclusions are presented below.

**Lemma** **2.** *Let $\Lambda \subseteq Z^{m}$ be a full-rank lattice and $\sigma \in R^{+}$ be a Gaussian parameter. Let $c_{i}\in Z^{m}$ and let $\alpha_{i}$ be mutually independent samples drawn from $D_{\Lambda +c_{i},s}$, for i=1,2,⋯,k. Let $b=\left(b_{1},b_{2},\cdots ,b_{k}\right)\in Z^{k}$, and define $g:=gcd(b_{1},b_{2},\cdots ,b_{k})$, $c:=\sum_{i=1}^{k}b_{i}c_{i}$. Suppose that $\sigma >\|b\|\;\eta_{\epsilon }\left(\Lambda \right)$ for some negligible ϵ. Then, $z=\sum_{i=1}^{k}b_{i}\alpha_{i}$ is within negligible statistical distance of $D_{g\Lambda +c,\sigma \|b\|}$.*

**Lemma** **3** ([19])**.** *Let k=poly(m) be even, q=poly(m) satisfy $q>{\left(mk\right)}^{2}$, and let $\delta >\omega (\sqrt{\log m})$. Then the statistical distance between $D_{Z^{m},\delta }$ and $D_{Z^{m},\delta \sqrt{(k\pm 2)/k}}$ is negligible in m.*

### 2.3. Algorithms

In this section, we introduce the main algorithms used to construct a lattice-based FSLHS scheme, as well as the relevant concepts and properties of **SampleDom** involved in the security proof.

**Theorem** **1** ([35])**.** *For an odd q≥2 and integer m≥6nlogq, n>0. There exists a probabilistic polynomial-time (PPT) algorithm TrapGen(q,n) that outputs matrices $A\in Z_{q}^{n\times m},T_{A}\in Z^{m\times m}$ where $T_{A}$ is a basis of $\Lambda^{⊥}\left(A\right)$, $\|T_{A}\left\|\leq O\left(n\log q\right)\;\;\mathrm{and}\;\;\right\|\tilde{T_{A}}\|\;\leq \;O\left(\sqrt{n\log q}\right)$. Moreover, A is statistically close to uniform over $Z_{q}^{n\times m}$.*

**Theorem** **2** ([19])**.** *Let k be an odd integer with k=poly(m). Define the sets*

$$\begin{array}{cc} V & =\left\{\upsilon \in F_{2}^{2k}|∥\upsilon ∥=k-1\right\}\cup \left\{0\right\},\\ R & =\left\{\gamma \in F_{2}^{2k}|∥\gamma ∥=k\right\}\cup \left\{0\right\},\\ K & =\left\{\kappa \in F_{2}^{2k}|∥\kappa ∥=k+1\right\}\cup \left\{0\right\}. \end{array}$$

*The polynomial-time algorithm Decompose takes as input $m\in F_{2}^{2k}$ and outputs a pair of vectors (υ,γ) with υ∈V, γ∈R∪K, and if m≠0 then at least one of them is nonzero.*

In Ref. [36], a trapdoor extension algorithm is defined: if a matrix *F* is composed of the concatenation of several matrices, and a trapdoor for one of these matrices is known, then this algorithm can generate a trapdoor for the entire matrix *F*, with the norm of the resulting trapdoor being equal to that of the known matrix trapdoor. This algorithm is referred to as **ExtBasis**, and its specific definition is as follows:

**Theorem** **3.** *Let $F=[A_{0}∥A_{1}∥A_{2}]$ be the concatenation of three matrices $A_{0},A_{1},A_{2}\in Z_{q}^{n\times m}$. If $T_{A_{i}}$ is a basis of $\Lambda_{q}^{⊥}\left(A_{i}\right)$, where i∈{0,1,2}, then there exists a deterministic polynomial-time algorithm $\mathrm{ExtBasis}(F,T_{A_{i}})$ that outputs a basis $T_{F}$ of $\Lambda_{q}^{⊥}\left(F\right)$, satisfying $∥\tilde{T_{F}}∥=∥\tilde{T_{A_{i}}}∥$.*

**Theorem** **4** ([37])**.** *Let m-dimensional lattice* Λ *and 0<ϵ<1. Matrix $T_{B}$ is a short basis for $\Lambda^{⊥}\left(B\right)$, $s\geq \|\tilde{T_{B}}\|\omega \left(\sqrt{\log m}\right)$ and $u\in Z^{n}$. There exists a PPT algorithm $\mathrm{SamplePre}(B,T_{B},u,s)$ that returns a vector $x\in \Lambda_{q}^{u}$ sampled from a distribution statistically close to $D_{\Lambda_{q}^{u}\left(B\right),s}$ whenever $\Lambda_{q}^{u}\left(B\right)$ is not empty.*

$\mathrm{SampleDom}(1^{m},s)$ is an algorithm that can sample from the distribution $D_{Z^{m},s}$ on $Z^{m}$, where $s\geq \omega (\sqrt{\log m})$. That is, if h is output by $\mathrm{SampleDom}(1^{m},s)$, then h is statistically close to a sample from $D_{Z^{m},s}$.

**Theorem** **5** ([37])**.** *Let q be a prime, m≥2nlogq be an integer, and the parameter $s\geq \omega (\sqrt{\log m})$. Then for all but a 2$q^{-n}$ fraction of $A\in Z_{q}^{n\times m}$, and for $h\leftarrow \mathrm{SampleDom}(1^{m},s)$, the vector α=A·hmodq follows a distribution that is statistically close to the uniform one over $Z_{q}^{n}$. Moreover, given **α**, the conditional distribution of h is $D_{\Lambda_{q}^{\alpha }\left(A\right),s}$.*

## 3. The FSLHS Scheme: Formal Definition and Security Model

In this section, we first present the formal definition of the FSLHS scheme, detailing its five constituent algorithms. We then proceed to construct its security model through a game-based experiment.

### 3.1. Formal Definition of FSLHS

FSLHS schemes employ a one-way key evolution mechanism, dividing the key lifecycle into multiple time periods, where each period uses a distinct key for signing. Keys are updated forward through one-way transformations, with old keys securely deleted, while the public key remains unchanged throughout. This design ensures that even if the current secret key is leaked, an attacker cannot forge signatures from previous time periods. This scheme includes five algorithms: **Setup**, **KeyUpdate**, **Sign**, **Combine**, **Verify**. The specific definition of the scheme is as follows.

**Definition** **4.** *An FSLHS scheme is a tuple of probabilistic polynomial-time algorithms (**Setup**, **KeyUpdate**, **Sign**, **Combine**, **Verify**), as follows:*

*$\left(pp,pk,sk_{0}\right)⟵\mathrm{Setup}\left(n,T\right)$: Taking the security parameter n and T time periods as input, where $T=2^{d}$ for some d∈N, it generates the public parameters pp, public key pk, and initial private key $sk_{0}$. The public parameters pp define the message space M, signature space S, tag space T, and the maximum number L of messages that are allowed to be combined under a single tag.*

*$\left(sk_{t+1}\right)⟵\mathrm{Keyupdate}\left(t,sk_{t}\right)$: Takes the time period t∈{0,1,2,⋯,T−1} and $sk_{t}$ for that period as input, generates $sk_{t+1}$ for time period t+1, and finally deletes the y $sk_{t}$ from time period t.*

*$\left(\sigma_{i}\right)⟵\mathrm{Sign}\left(pp,pk,t,sk_{t},\tau ,m_{i}\right)$: Input the pp, the pk, the time period t, the $sk_{t}$ for that period, a tag τ∈T, and a message $m_{i}$ from the message subspace V labeled by τ, where V⊂M. Finally, output the signature $\sigma_{i}\in S$ for message $m_{i}$ in time period t.*

*$\left(\sigma \right)⟵\mathrm{Combine}(pp,t,\tau ,{\left\{\left(c_{i},\sigma_{i}\right)\right\}}_{i=1}^{l})$: Input the pp, the time period t, the tag τ, and a set of tuples ${\left\{\left(c_{i},\sigma_{i}\right)\right\}}_{i=1}^{l}$, where $c_{i}\in \left\{0,1\right\}$, l<L, and $\sigma_{i}\in S$ is the signature of $m_{i}\in M$ at time period t. This algorithm generates the signature $\sigma =\sum_{i=1}^{l}c_{1}\sigma_{i}\in S$ for $m=\sum_{i=1}^{l}c_{i}m_{i}\in M$ at time period t.*

*(0or1)⟵Verify(pk,t,τ,m,σ): Input the pk, the time period t, the tag τ∈T, the message m from V⊂M it identifies, and the signature σ∈S. If the signature σ is valid, output “1” (accept). Otherwise, output “0” (reject).*

*Correctness: The FSLHS scheme requires that two types of signatures can be accepted by the verification algorithm: the original individual signature $\sigma_{i}\in S$, and the combined signature σ∈S generated by the combination algorithm. The specific verification process is as follows:*

*(1)*  *For any τ and any message $m_{i}$ from the subspace V⊂M it identifies, if the signature $\sigma_{i}⟵\mathrm{Sign}\left(pp,pk,t,sk_{t},\tau ,m_{i}\right)$ at time period t, then the verification algorithm satisfies*
$$1⟵\mathrm{Verify}(pk,t,\tau ,m_{i},\sigma_{i}).$$
*(2)*  *If the signature $\sigma_{i}⟵\mathrm{Sign}\left(pp,pk,t,sk_{t},\tau ,m_{i}\right)$ at time period t, then the verification algorithm satisfies*
$$1⟵\mathrm{Verify}(pk,t,\tau ,m=\sum_{i=1}^{l}c_{i}m_{i},\mathrm{Combine}\left(pp,t,\tau ,{\left\{\left(c_{i},\sigma_{i}\right)\right\}}_{i=1}^{l}\right)).$$

**Figure 1:**
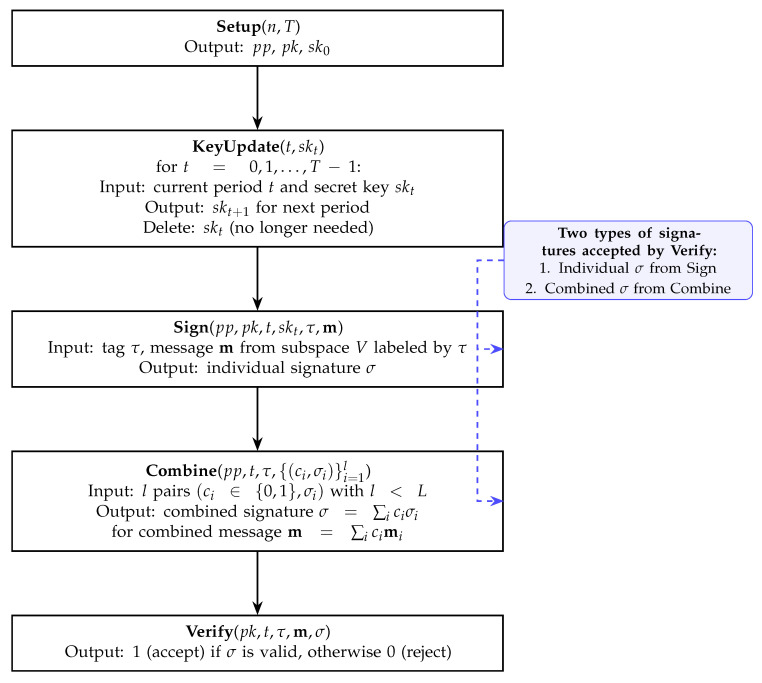
Flow of the five algorithms in the FSLHS scheme.

To more intuitively demonstrate the logical relationships, execution order, and data flow among the five algorithms mentioned above (**Setup**, **KeyUpdate**, **Sign**, **Combine**, **Verify**), Figure 1 presents a schematic diagram of the overall workflow of the FSLHS scheme.

### 3.2. Security Model of FSLHS

The security model for FSLHS schemes is derived from the standard LHS security model by incorporating forward security requirements. Specifically, this model mandates that even after an adversary performs a series of queries, if the adversary outputs a forged tuple $\left(m^{*},t^{*},\tau^{*},\sigma^{*}\right)$ containing the message $m^{*}$, time period $t^{*}<T$, tag $\tau^{*}$, and signature $\sigma^{*}$, the advantage of the adversary in making the verification algorithm **Verify**$\left(pk,m^{*},t^{*},\tau^{*},\sigma^{*}\right)$ output “1” must be negligible. This security property is typically formalized through an interactive game between the adversary A and the challenger C, with the detailed definition as follows:

**Definition** **5** (Forward-secure unforgeability)**.** *If for any PPT adversary A, the advantage of winning the following game under the security parameter n is negligible, then the scheme satisfies forward-secure unforgeability.*

**Setup**: A sends the security parameter *n* to C. C obtains the public parameters pp, the public key pk, and the initial secret key $sk_{0}$ by executing the **Setup**. Finally, C sends pp and pk to A, while keeping $sk_{0}$ confidential.

**Queries**: A can adaptively make a polynomial number of signature queries in any time period *t*. When A wishes to advance to the next period, it submits a key update query to C and obtains the key $sk_{t+1}$ for the time period t+1. Note that once A obtains $sk_{t+1}$, it can no longer make any queries for previous periods. A may choose to stop querying at any time by submitting a break-in time $\bar{t}\leq T-1$, thereby initiating a break-in query. After that, A is not allowed to make any further queries. The specific response process of C to all the above queries is as follows:

**Keyupdate queries**: For time period *t*, A sends *t* to C. C checks whether t<T−1 holds. If it does, C executes the $\mathrm{Keyupdate}(t,sk_{t})$ to obtain the key $sk_{t+1}$ corresponding to t+1 and returns it to A. Otherwise, C returns ⊥, indicating that the update has failed.

**Sign queries**: A submits to C the time period t<T−1 and the basis vectors $m_{1},\ldots ,m_{k}$ of the message subspace *V* to be signed. Upon receiving *V*, C selects a tag $\tau \overset{\$}{⟵}{\left\{0,1\right\}}^{k}$, then generates signatures $\sigma_{i}$ by executing the $\mathrm{Sign}(pp,t,sk_{t},\tau ,m_{i})$ for i=1,2,…,k and returns the set of signatures ${\left\{\sigma_{i}\right\}}_{i=1}^{k}$ to A.

**Break-in query**: A selects and sends a break-in time $\bar{t}$ (satisfying $\bar{t}<T-1$) to C. C returns the corresponding secret key $sk_{\bar{t}}$ for time period $\bar{t}$ to A. After this, the game immediately enters the output phase. That is to say, apart from being allowed to output only one forgery, A is not permitted to make any signature or key update queries.

**Forgery**: A outputs a forged tuple $\left(t^{\prime },\tau^{\prime },m^{\prime },\sigma^{\prime }\right)$, where the time period satisfies $t^{\prime }<T-1$, $\tau^{\prime }$ is a tag, the message $m^{\prime }\in V^{\prime }$ labeled by $\tau^{\prime }$, and $\sigma^{\prime }$ is the signature for $m^{\prime }$. A is said to win the game if the forgery satisfies the following conditions:

(1) For time period $t^{\prime }<\bar{t}$, the tuple $\left(t^{\prime },\tau^{\prime },m^{\prime }\right)$ has never been queried for a signature.
(2) The signature verification result satisfies $1\leftarrow \mathrm{Verify}(pk,t^{\prime },\tau^{\prime },m^{\prime },\sigma^{\prime })$.
(3) It conforms to one of the following two types:
  (a) Type 1: For all signature queries involving a tag τ in time period $t^{\prime }$, $\tau^{\prime }\neq \tau$.
  (b) Type 2: There exists some τ such that $\tau^{\prime }=\tau$, but $m^{\prime }\notin V$, where *V* denotes the subspace spanned by the vectors ${\left\{m_{i}\right\}}_{i=1}^{k}$ and is labeled by τ in time period $t^{\prime }$.

**Figure 2:**
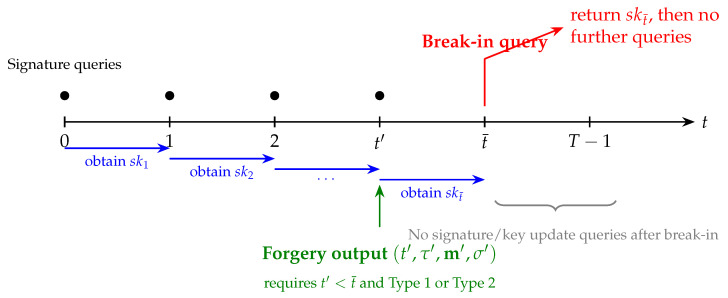
Schematic diagram of the query timeline in the forward-secure unforgeability game.

The above security game involves multiple time periods and various types of adversary queries (**Keyupdate queries**, **Sign queries**, **Break-in query**). The temporal logic among these directly determines the constraints of forward-secure unforgeability. To more intuitively illustrate the relationship between these queries and time periods, we present a timeline diagram in Figure 2. In the figure, the black dots above the time axis represent signature queries for the corresponding time periods, and the blue arrows below the time axis indicate the signature queries for those time periods.

## 4. Lattice-Based FSLHS Scheme

In this section, we primarily focus on the foundation and core aspects of the scheme construction. First, we provide a detailed explanation of the correspondence between the leaf nodes of the binary tree and the time periods, and elaborate on how to construct the corresponding matrix and generate its trapdoor based on the binary representation of the nodes, in preparation for the key update algorithm. Subsequently, we present the specific design of the lattice-based FSLHS scheme and provide a complete proof of its correctness and security.

### 4.1. Time Periods on a Binary Tree

**Figure 3:**
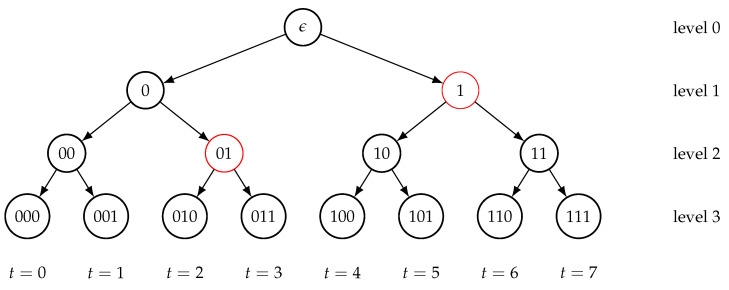
A binary tree with depth d=3, i.e., having $T=2^{3}$ time periods.

Inspired by [30], consider a complete binary tree of depth *d*. We assign the time periods $t\in \{0,1,\ldots ,2^{d}-1\}$ to its leaf nodes, arranging them from left to right in increasing order. For a given time period *t*, there is a unique path $t=(t_{1},\ldots ,t_{d})$ from the root node ϵ to the corresponding leaf node, where at each level i∈[d], $t_{i}=0$ indicates a left branch and $t_{i}=1$ indicates a right branch (as shown in Figure 3). Thus, for binary tree nodes with depth less than *d*, where i≠d, a node $\nu^{\left(i\right)}$ at level *i* can be described by a unique binary bit string $\nu^{\left(i\right)}=\left(\nu_{1},\ldots ,\nu_{i}\right)$ corresponding to the path from the root to that node, where $\nu_{i}\in \left\{0,1\right\}$. This means that for a node $\nu^{\left(i\right)}=\left(\nu_{1},\ldots ,\nu_{i}\right)$, we can construct the corresponding matrix $F_{\nu^{\left(i\right)}}=\left[A_{0}∥A_{1}^{\left(\nu_{1}\right)}∥\ldots ∥A_{i}^{\left(\nu_{i}\right)}\right]$. Correspondingly, for a leaf node (the time period $t=(t_{1},\ldots ,t_{d})$), we can construct the matrix $F_{t}=\left[A_{0}∥A_{1}^{\left(t_{1}\right)}∥\ldots ∥A_{d}^{\left(t_{d}\right)}\right]$. Here, $A_{0}$ and its associated trapdoor $T_{A_{0}}$ are generated by the **TrapGen**, and for all i∈[d] and b∈{0,1}, the matrices $A_{i}^{\left(b\right)}$ are chosen uniformly at random.

Next, we describe the process of generating leaf node trapdoors from the root node trapdoor. Let $A_{0}$ be the matrix corresponding to the root node, and let $T_{A_{0}}$ be the trapdoor of $\Lambda^{⊥}\left(A_{0}\right)$. Each node $\nu^{\left(i\right)}=\left(\nu_{1},\cdots ,\nu_{i}\right)$ corresponds to the matrix $F_{\nu^{\left(i\right)}}$. By inputting $F_{\nu^{\left(i\right)}}$ and $T_{A_{0}}$ into the **ExtBasis**, we can obtain the trapdoor $T_{\nu^{\left(i\right)}}$ of $\Lambda^{⊥}\left(F_{\nu^{\left(i\right)}}\right)$, i.e.,

$$T_{\nu^{\left(i\right)}}\leftarrow \mathrm{ExtBasis}\left(F_{\nu^{\left(i\right)}},T_{A_{0}}\right),\;\mathrm{where}\;F_{\nu^{\left(i\right)}}=\left[A_{0}∥A_{1}^{\left(\nu_{1}\right)}∥A_{2}^{\left(\nu_{2}\right)}∥\cdots ∥A_{i}^{\left(\nu_{i}\right)}\right].$$

If the trapdoor $T_{\nu^{\left(k\right)}}$ of any ancestor node $\nu^{\left(k\right)}$ (k<i) of node $\nu^{\left(i\right)}$ is known, then $T_{\nu^{\left(i\right)}}$ can be derived. Let $\nu^{\left(i\right)}=\left(\nu_{1},\cdots ,\nu_{k},\nu_{k+1},\cdots ,\nu_{i}\right)$, then we have

$$T_{\nu^{\left(i\right)}}\leftarrow \mathrm{ExtBasis}\left(F_{\nu^{\left(i\right)}},T_{\nu^{\left(k\right)}}\right),\;\mathrm{where}\;F_{\nu^{\left(i\right)}}=\left[A_{0}∥A_{1}^{\left(\nu_{1}\right)}∥\cdots ∥A_{k}^{\left(\nu_{k}\right)}∥\cdots ∥A_{i}^{\left(\nu_{i}\right)}\right].$$

Therefore, as long as the trapdoor of any ancestor node of a given time period (corresponding to a leaf node) is known, the trapdoor for that time period can be derived.

### 4.2. Design of Our Scheme

As described in Section 3.1, the FSLHS scheme typically consists of the Setup, Keyupdate, Sign, Combine, and Verify algorithms. The lattice-based FSLHS scheme proposed in this paper also follows this framework, with the specific definitions of each algorithm as follows.

Setup(n,T): Taking the security parameter *n* and the total number of time periods $T=2^{d}$ (where *d* is the depth of the binary tree) as inputs, the Key Generation Center (KGC) selects the following parameters: *L* denotes the maximum number of signatures that can be combined, an even integer k=poly(n), an integer m=⌈6nlogq⌉, a prime number $q>{\left(Lmk\right)}^{2}$, a Gaussian parameter $\delta \geq \sqrt{2km\log q}\log m$, a tag space $T={\left\{0,1\right\}}^{k}$, a message space $M=F_{2}^{k}$, and a signature space $S=Z^{(d+1)m}$. The KGC then performs the following operations:

(1) Randomly select *k* vectors $a_{1},a_{2},\cdots ,a_{k}$ from $Z_{q}^{n}$.
(2) Randomly select 2d matrices $A_{1}^{\left(0\right)},A_{1}^{\left(1\right)},A_{2}^{\left(0\right)},A_{2}^{\left(1\right)},\cdots ,A_{d}^{\left(0\right)},A_{d}^{\left(1\right)}$ from $Z_{q}^{n\times m}$.
(3) Execute the **TrapGen**(n,m,q) to obtain a pair of matrices $\left(A_{0},T_{A_{0}}\right)$, where $A_{0}$ is the matrix for the root node and $T_{A_{0}}$ is its associated trapdoor matrix.
(4) Output the public parameters $pp=\{n,m,q,d,k,L,\delta ,T,M,S,{\left\{a_{j}\right\}}_{j=1}^{k},A_{1}^{\left(0\right)},A_{1}^{\left(1\right)},A_{2}^{\left(0\right)},$ $A_{2}^{\left(1\right)},\cdots ,A_{d}^{\left(0\right)},A_{d}^{\left(1\right)}\}$, the public key $pk=A_{0}$, and the initial secret key ${sk}_{\epsilon }=T_{A_{0}}$.

$\mathrm{Keyupdate}(pp,pk,t,st_{t})$: Given the public parameters pp, the time period *t*, the secret key $sk_{t}$ for this time period, and the public key pk, the signer executes this algorithm to generate the secret key $sk_{t+1}$ for the next time period t+1. The specific key update process is as follows.

For any leaf node *t*, define its minimal cover set Node(t) as the smallest set of nodes that satisfies the following condition: the set contains all ancestors of the leaves in {t,…,T−1}, but does not contain any ancestor of the leaves in {0,…,t−1}. For example, in Figure 3, Node(0)={ϵ}, Node(1)={001,01,1}, Node(2)={01,1} (i.e., two red circles in Figure 3), Node(3)={011,1}, Node(4)={1}, Node(5)={101,11}, Node(6)={11}, and Node(7)={111}.

The secret key $sk_{t}$ at time period *t* is defined as the set of trapdoors corresponding to all nodes in Node(t), i.e., $sk_{t}=\left\{T_{\nu }|\nu \in \mathrm{Node}\left(t\right)\right\}$. The key to this construction is the ExtBasis algorithm. If the trapdoor $T_{\mathrm{anc}}$ of an ancestor node anc is known, then the trapdoor $T_{\mathrm{desc}}$ of any descendant node desc can be derived, because the matrix $F_{\mathrm{desc}}$ is a concatenation of $F_{\mathrm{anc}}$ with additional random matrices (see Section 4.1 for details). This one-way derivability ($T_{\mathrm{anc}}\to T_{\mathrm{desc}}$) enables forward security: Node(t) contains the “latest” ancestor trapdoors that suffice to generate all necessary leaf trapdoors for time period *t* and future periods, while older trapdoors (which could have been compromised) are discarded. Consequently, the minimal cover set provides exactly the minimal set of trapdoors required for the current and all subsequent time periods.

The secret key $sk_{t}$ at time period *t* contains the trapdoors corresponding to all nodes in Node(t). Taking Figure 3 as an example, we have $sk_{0}=sk_{\epsilon }=\left\{T_{A_{0}}\right\}$, $sk_{1}=\left\{T_{001},T_{01},T_{1}\right\}$, where $T_{001}$, $T_{01}$, and $T_{1}$ are the trapdoors associated with the matrices $F_{001}=[A_{0}∥A_{1}^{\left(0\right)}∥A_{2}^{\left(0\right)}∥A_{3}^{\left(1\right)}]$, $F_{01}=[A_{0}∥A_{1}^{\left(0\right)}∥A_{2}^{\left(1\right)}]$, and $F_{1}=\left[A_{0}∥A_{1}^{\left(1\right)}\right]$, respectively.

To update the secret key from $sk_{t}$ to $sk_{t+1}$, the signer first determines the minimal cover set Node(t+1). Then, using the trapdoors in $sk_{t}$ (as described in Section 4.1), the signer derives the trapdoors for all nodes in Node(t+1)∖Node(t). Finally, the signer deletes all trapdoors corresponding to the nodes in Node(t)∖Node(t+1). For instance, since Node(2)∖Node(1)={01,1} and Node(1)∖Node(2)={001}, it follows that $sk_{2}=\left\{T_{01},T_{1}\right\}$.

$\mathrm{Sign}(pp,\tau ,t,sk_{t},m_{i})$: The signer takes as input the pp, the time period *t* along with its corresponding key $sk_{t}$, a tag $\tau =(\tau_{1},\tau_{2},\cdots ,\tau_{k})\in T$, and the subspace V⊂M tagged by τ whose basis vectors are $m_{1},\cdots ,m_{k}$. For each basis vector $m_{i}=\left(m_{i1},m_{i2},\cdots ,m_{ik}\right)\;\left(1\leq i\leq k\right)$, the signer performs the following operations:

(1) Construct the matrix $F_{t}=[A_{0}∥A_{1}^{\left(t_{1}\right)}∥\cdots ∥A_{d}^{\left(t_{d}\right)}]$ according to the time period $t=(t_{1},\cdots ,t_{d})$.
(2) Check whether the trapdoor $T_{t}$ corresponding to $F_{t}$ is contained in $sk_{t}$. If not, extract the trapdoor corresponding to the ancestor nodes of *t* from $sk_{t}$ and invoke the **ExtBasis** to generate $T_{t}$ (this is because not all trapdoors corresponding to leaf nodes are directly stored in $sk_{t}$. For example, when t=2, its binary representation is (010), while the corresponding trapdoor $T_{010}$ is not in $sk_{2}=\left\{T_{01},T_{1}\right\}$).
(3) Execute the $\mathrm{Decompose}(m_{i})$ to obtain a pair of vectors $\left(\upsilon_{i},\gamma_{i}\right)$, where $m_{i}=\left(m_{i1},\cdots ,m_{ik}\right)=\upsilon_{i}+\gamma_{i}$, $\upsilon_{i}=\left(\upsilon_{i1},\ldots ,\upsilon_{ik}\right)$ and $\gamma_{i}=\left(\gamma_{i1},\ldots ,\gamma_{ik}\right)$.
(4) Determine whether there exists a zero vector in $\left(\upsilon_{i},\gamma_{i}\right)$. If so, compute
  $$h_{i}=\sum_{j=1}^{k}{\left(-1\right)}^{\tau_{j}}m_{ij}a_{j},$$
  and invoke the $\mathrm{SamplePre}(F_{t},T_{t},h_{i},\delta )$ to output the signature $\sigma_{i}$. Otherwise, compute
  $$h\left(\upsilon_{i}\right)=\sum_{j=1}^{k}{\left(-1\right)}^{\tau_{j}}\upsilon_{ij}a_{j}\;\mathrm{and}\;h\left(\gamma_{i}\right)=\sum_{j=1}^{k}{\left(-1\right)}^{\tau_{j}}\gamma_{ij}a_{j},$$
  then invoke
  $$\mathrm{SamplePre}\left(F_{t},T_{t},h\left(\upsilon_{i}\right),\delta \right)\;\mathrm{and}\;\mathrm{SamplePre}\left(F_{t},T_{t},h\left(\gamma_{i}\right),\delta \right)$$
  respectively to obtain $\sigma (\upsilon_{i})$ and $\sigma (\gamma_{i})$, and finally output
  $$\sigma_{i}=\sigma \left(\upsilon_{i}\right)+\sigma \left(\gamma_{i}\right).$$
(5) Regard $\sigma_{i}$ as the signature of the vector $m_{i}$ within time period *t*.

$\mathrm{Combine}(pp,\tau ,t,{\left\{\left(c_{i},\sigma_{i}\right)\right\}}_{i=1}^{l})$: Given the pp, the time period *t*, the tag $\tau =\left(\tau_{1},\tau_{2},\cdots ,\tau_{k}\right)\in {\left\{0,1\right\}}^{k}$, and a tuple set ${\left\{\left(c_{i},\sigma_{i}\right)\right\}}_{i=1}^{l}$, where l≤L, $c_{i}\in \left\{0,1\right\}$ and each $\sigma_{i}$ is obtained via $\mathrm{Sign}(pp,\tau ,t,sk_{t},m_{i})$, the algorithm outputs the signature $\sigma =\sum_{i=1}^{l}c_{i}\sigma_{i}$ for the message $m=\sum_{i=1}^{l}c_{i}m_{i}$ in time period *t*.

$\mathrm{Verify}(t,\tau ,pk_{t},m,\sigma )$: Given the time period *t*, public key $pk_{t}$, a tag $\tau =\left(\tau_{1},\tau_{2},\cdots ,\tau_{k}\right)\in {\left\{0,1\right\}}^{k}$, a vector m∈V, and a signature σ, the verification process is as follows:

(1) Compute $h=\sum_{j=1}^{l}c_{j}h_{j}$, where $h_{j}=\sum_{i=1}^{k}{\left(-1\right)}^{\tau_{i}}m_{ji}a_{i}$.
(2) If the following conditions are satisfied:
  (a) $F_{t}\sigma \;\mathrm{mod}\;q=h$;
  (b) $∥\sigma ∥<2L\delta \sqrt{2k(d+1)m}$, then the algorithm outputs “1”. Otherwise, it outputs “0”.

**Note**: Since the methods for handling correctness and security proofs are the same whether the vector decomposition of the message $m_{i}$ into a pair of vectors results in one zero vector or no zero vectors, without loss of generality, we only consider the case where $m_{i}$ is decomposed into two non-zero vectors in the subsequent analysis.

### 4.3. Correctness

We demonstrate that signatures generated by the signature algorithm and the combination algorithm in this scheme can all be successfully verified by the verification algorithm.

Verify the signature $\sigma_{i}$: For the signature $\sigma_{i}$ generated by the $\mathrm{Sign}(pp,\tau ,t,sk_{t},m_{i})$, according to Lemma 1 and Theorem 4, the following expressions hold:

$$∥\sigma_{i}∥\;=\;∥\sigma \left(\upsilon_{i}\right)+\sigma \left(\gamma_{i}\right)∥\;\leq \;∥\sigma \left(\upsilon_{i}\right)∥+∥\sigma \left(\gamma_{i}\right)∥\;\leq \;2\delta \sqrt{(d+1)m}\;\leq \;2L\delta \sqrt{2k(d+1)m}$$

and

$$\begin{array}{cc} F_{t}\sigma_{i}\;\mathrm{mod}\;q\; & =F_{t}\left(\sigma \left(\upsilon_{i}\right)+\sigma \left(\gamma_{i}\right)\right)\;\mathrm{mod}\;q=h\left(\upsilon_{i}\right)+h\left(\gamma_{i}\right)\\ \; & =\sum_{j=1}^{k}{\left(-1\right)}^{\tau_{j}}\upsilon_{ij}a_{j}+\sum_{j=1}^{k}{\left(-1\right)}^{\tau_{j}}\gamma_{ij}a_{j}\\ \; & =\sum_{j=1}^{k}{\left(-1\right)}^{\tau_{j}}\left(\upsilon_{ij}+\gamma_{ij}\right)a_{j}\\ \; & =\sum_{j=1}^{k}{\left(-1\right)}^{\tau_{j}}m_{ij}a_{j}=h_{i}. \end{array}$$

Thus, the output result of the Sign passes the verification performed by the Verify.

Verify the combined signature σ: Given a time period *t*, a tag τ, and a tuple set ${\left\{\left(c_{i},\sigma_{i}\right)\right\}}_{i=1}^{l}$, where l≤L,$c_{i}\in \left\{0,1\right\}$ and $\sigma_{i}$ generated by the $\mathrm{Sign}(pp,\tau ,t,sk_{t},m_{i})$, it is required to prove that $\mathrm{Verify}(t,\tau ,pk_{t},m=\sum_{i=1}^{l}c_{i}m_{i},\mathrm{Combine}\left(pp,\tau ,t,{\left\{\left(c_{i},\sigma_{i}\right)\right\}}_{i=1}^{l}\right))$ outputs “1”. According to the **Combine**, $\sigma =\sum_{i=1}^{l}c_{i}\sigma_{i}$ is a signature for the message $m=\sum_{i=1}^{l}c_{i}m_{i}$ output by the algorithm. Hence the following holds:

$$\begin{array}{c} ∥\sigma ∥\;=\;∥\sum_{i=1}^{l}c_{i}\sigma_{i}∥\;\leq \;2L\delta \sqrt{(d+1)m}\;\leq \;2L\delta \sqrt{2k(d+1)m}. \end{array}$$

Since $\sigma_{i}$ is generated by $\mathrm{sign}(pp,\tau ,t,sk_{t},m_{i})$, we have $F_{t}\sigma_{i}\;\mathrm{mod}\;q=h_{i}$. It then follows that

$$\begin{array}{c} F_{t}\sigma \;\mathrm{mod}\;q=F_{t}\sum_{i=1}^{l}c_{i}\sigma_{i}\;\mathrm{mod}\;q=\sum_{i=1}^{l}c_{i}F_{t}\sigma_{i}\;\mathrm{mod}\;q=\sum_{i=1}^{l}c_{i}h_{i}=h. \end{array}$$

Therefore, the output of $\mathrm{Verify}(t,\tau ,pk_{t},m=\sum_{i=1}^{l}c_{i}m_{i},\mathrm{Combine}\left(pp,\tau ,t,{\left\{\left(c_{i},\sigma_{i}\right)\right\}}_{i=1}^{l}\right))$ being “1” is proved. In conclusion, the proposed scheme satisfies correctness.

### 4.4. Forward-Secure Unforgeability

This section proves the security of our scheme by reducing it to the hardness of the SIS problem over lattices. Specifically, if there exists a PPT adversary that can forge a signature for a certain time period with a non-negligible advantage, then a challenger can solve an instance of the SIS problem with non-negligible probability. Hence, this demonstrates that our scheme satisfies forward-secure unforgeability.

**Theorem** **6** (Forward-secure unforgeability)**.** *If there exists a PPT adversary A that achieves a non-negligible advantage ε in breaking the unforgeability of the forward-secure signature scheme with a total of $T=2^{d}$ time periods, then we can construct a PPT algorithm C such that C solves the ${\mathrm{SIS}}_{q,n,(2d+1)m,\beta }$ problem with probability at least $\frac{(1-2^{-\omega (\log m)})\varepsilon }{T}$, where $\beta =2L\delta \sqrt{2k(d+1)m}$ and d is the depth of the binary tree.*

**Proof.**  Suppose C aims to solve an SIS instance Fv=0modq, where $F=[A_{0}∥U_{1}^{\left(0\right)}∥U_{1}^{\left(1\right)}∥U_{2}^{\left(0\right)}∥U_{2}^{\left(1\right)}∥\cdots ∥U_{d}^{\left(0\right)}∥U_{d}^{\left(1\right)}]\in Z_{q}^{n\times (2d+1)m}$ with $A_{0},U_{i}^{\left(b\right)}\in Z_{q}^{n\times m}$, i=1,2,⋯,d and b∈{0,1}.

**Setup**: First, C selects a target time period $t^{*}=\left(t_{1}^{*},t_{2}^{*},\ldots ,t_{d}^{*}\right)\leq T-1$, and A needs to guess this period. Therefore, A chooses a time period $t\overset{\$}{⟵}\left\{0,1,\ldots ,T-1\right\}$. Hence, the probability that A correctly guesses $t^{*}$ is 1/T. Then C obtains the secret key through the following procedure:

(1) For each i∈[d], C sets $A_{i}^{\left(t_{i}^{*}\right)}=U_{i}^{\left(t_{i}^{*}\right)}$. For $b\neq t_{i}^{*}$ and b∈{0,1}, C executes the TrapGen to obtain a pair of matrices $\left(A_{i}^{\left(b\right)},T_{A_{i}^{\left(b\right)}}\right)$, where $T_{A_{i}^{\left(b\right)}}$ is the trapdoor of $\Lambda_{q}^{⊥}\left(A_{i}^{\left(b\right)}\right)$.
(2) C invokes the $\mathrm{SampleDom}(1^{(d+1)m},\delta \sqrt{\frac{2}{k}})$ to generate *k* vectors $v_{1},v_{2},\cdots ,v_{k}$, and computes $a_{j}=F_{t^{*}}v_{j}\;\mathrm{mod}\;q$ for j=1,…,k, where $F_{t^{*}}=[A_{0}∥A_{1}^{\left(t_{1}^{*}\right)}∥A_{2}^{\left(t_{2}^{*}\right)}∥\cdots ∥A_{d}^{\left(t_{d}^{*}\right)}]$.

Finally, C sends the public key $pk=(A_{0},A_{1}^{\left(0\right)},A_{1}^{\left(1\right)},A_{2}^{\left(0\right)},A_{2}^{\left(1\right)},\cdots ,A_{d}^{\left(0\right)},A_{d}^{\left(1\right)},a_{1},\ldots ,a_{k})$ to A and keeps $T_{A_{i}^{\left(b\right)}}$ confidential.

**Queries**: The adaptive query process of A and the response process of C are as follows:

**Keyupdate queries**: A sends the time period $t=(t_{1},t_{2},\cdots ,t_{d})$ to C. If $t\leq t^{*}$, C aborts the query. If $t>t^{*}$, C finds the smallest index h<d such that $t_{h}\neq t_{h}^{*}$. Then, C generates the trapdoor $T_{t^{\left(h\right)}}$ associated with node $t^{\left(h\right)}$ by the $\mathrm{ExtBasis}(B∥A_{h}^{\left(t_{h}\right)},T_{A_{h}^{\left(t_{h}\right)}})$, where $B=[A_{0}∥A_{1}^{\left(t_{1}\right)}∥A_{2}^{\left(t_{2}\right)}∥\cdots ∥A_{h-1}^{\left(t_{h-1}\right)}]$, and $T_{A_{h}^{\left(t_{h}\right)}}$ is the trapdoor of $\Lambda_{q}^{⊥}\left(A_{h}^{\left(t_{h}\right)}\right)$. Subsequently, C computes all trapdoors in $sk_{t}$ by following the exact same key update procedure as in the actual scheme.

**Sign queries**: A submits to C a time period $t=(t_{1},t_{2},\cdots ,t_{d})$ and a set of basis vectors ${\left\{m_{j}^{\left(i\right)}\right\}}_{j=1}^{k}$ for the message subspace $V^{\left(i\right)}$, where i<Q and *Q* is the number of signature queries. Upon receiving them, C first selects a tag $\tau^{\left(i\right)}=\left(\tau_{1}^{\left(i\right)},\tau_{2}^{\left(i\right)},\cdots ,\tau_{k}^{\left(i\right)}\right)\overset{\$}{⟵}{\left\{0,1\right\}}^{k}$ and returns it to A, then proceeds with the following operations:

(1) Construct $F_{t}=[A_{0}∥A_{1}^{\left(t_{1}\right)}∥\cdots ∥A_{d}^{\left(t_{d}\right)}]$.
(2) Check whether $t\neq t^{*}$ holds.

Case 1: If it holds, C performs the following steps:

(a) Obtain $T_{t}$ via $\mathrm{ExtBasis}(F_{t},T_{A_{h}^{\left(t_{h}\right)}})$, where h≤d is the smallest index such that $t_{h}\neq t_{h}^{*}$.
(b) Execute $\mathrm{Decompose}(m_{j}^{\left(i\right)})$ to obtain a pair of vectors $\left(\upsilon_{j}^{\left(i\right)},\gamma_{j}^{\left(i\right)}\right)$, where $m_{j}^{\left(i\right)}=\left(m_{j1}^{\left(i\right)},\cdots ,m_{jk}^{\left(i\right)}\right)=\upsilon_{j}^{\left(i\right)}+\gamma_{j}^{\left(i\right)}$, $\upsilon_{j}^{\left(i\right)}=\left(\upsilon_{j1}^{\left(i\right)},\ldots ,\upsilon_{jk}^{\left(i\right)}\right)$ and $\gamma_{j}^{\left(i\right)}=\left(\gamma_{j1}^{\left(i\right)},\ldots ,\gamma_{jk}^{\left(i\right)}\right)$.
(c) Compute $h\left(\upsilon_{j}^{\left(i\right)}\right)=\sum_{u=1}^{k}{\left(-1\right)}^{\tau_{u}^{\left(i\right)}}\upsilon_{ju}^{\left(i\right)}a_{u}\;\mathrm{and}\;h\left(\gamma_{j}^{\left(i\right)}\right)=\sum_{u=1}^{k}{\left(-1\right)}^{\tau_{u}^{\left(i\right)}}\gamma_{ju}^{\left(i\right)}a_{u}$, then invoke $\mathrm{SamplePre}\left(F_{t},T_{t},h\left(\upsilon_{j}^{\left(i\right)}\right),\delta \right)\;\mathrm{and}\;\mathrm{SamplePre}\left(F_{t},T_{t},h\left(\gamma_{j}^{\left(i\right)}\right),\delta \right)$ respectively to obtain $\sigma (\upsilon_{j}^{\left(i\right)})$ and $\sigma (\gamma_{j}^{\left(i\right)})$, and set $\sigma_{j}^{\left(i\right)}=\sigma \left(\upsilon_{j}^{\left(i\right)}\right)+\sigma \left(\gamma_{j}^{\left(i\right)}\right)$ for j=1,2,⋯,k.
(d) Output ${\left\{\sigma_{j}^{\left(i\right)}\right\}}_{j=1}^{k}$ and send $\left(\tau^{\left(i\right)},{\left\{\sigma_{j}^{\left(i\right)}\right\}}_{j=1}^{k}\right)$ in time period *t* to A.

Case 2: If it does not hold, the operation of C is as follows:

(a) Execute $\mathrm{Decompose}(m_{j}^{\left(i\right)})$ to obtain a pair of vectors $\left(\upsilon_{j}^{\left(i\right)},\gamma_{j}^{\left(i\right)}\right)$, where $m_{j}^{\left(i\right)}=\left(m_{j1}^{\left(i\right)},\ldots ,m_{jk}^{\left(i\right)}\right)=\upsilon_{j}^{\left(i\right)}+\gamma_{j}^{\left(i\right)}$, $\upsilon_{j}^{\left(i\right)}=\left(\upsilon_{j1}^{\left(i\right)},\ldots ,\upsilon_{jk}^{\left(i\right)}\right)$ and $\gamma_{j}^{\left(i\right)}=\left(\gamma_{j1}^{\left(i\right)},\ldots ,\gamma_{jk}^{\left(i\right)}\right)$.
(b) Compute $\sigma \left(\upsilon_{j}^{\left(i\right)}\right)=\sum_{u=1}^{k}{\left(-1\right)}^{\tau_{u}^{\left(i\right)}}\upsilon_{ju}^{\left(i\right)}v_{u}$ and $\sigma \left(\gamma_{j}^{\left(i\right)}\right)=\sum_{u=1}^{k}{\left(-1\right)}^{\tau_{u}^{\left(i\right)}}\gamma_{ju}^{\left(i\right)}v_{u}$. Set $\sigma_{j}^{\left(i\right)}=\sigma \left(\upsilon_{j}^{\left(i\right)}\right)+\sigma \left(\gamma_{j}^{\left(i\right)}\right)$ for j=1,2,⋯,k.
(c) Output ${\left\{\sigma_{j}^{\left(i\right)}\right\}}_{j=1}^{k}$ and send $\left(\tau^{\left(i\right)},{\left\{\sigma_{j}^{\left(i\right)}\right\}}_{j=1}^{k}\right)$ in time period *t* to A.

**Break-in query**: A sends the time period *t* to C. Upon receiving *t*, C first checks whether $t\leq t^{*}$ holds. If so, C aborts the query. Otherwise, C sets *t* as the break-in time $\bar{t}$. Then, following the same procedure as in a key-update query, C generates the secret key $sk_{\bar{t}}$ in time period $\bar{t}$ and returns it to A.

**Forgery**: After the query phase, A outputs a forgery tuple $\left(\tau^{\prime },t^{\prime },m^{\prime },\sigma^{\prime }\right)$. C first checks whether $t^{\prime }=t^{*}$ holds. If not, C aborts the game. Otherwise, C accepts the forgery. For the tuple $\left(\tau^{\prime },t^{\prime },m^{\prime },\sigma^{\prime }\right)$, it satisfies $F_{t^{\prime }}\sigma^{\prime }\;\mathrm{mod}\;q=h^{\prime }$ and $∥\sigma^{\prime }∥<2L\delta \sqrt{2k(d+1)m}$, where $F_{t^{\prime }}=[A_{0}∥A_{1}^{\left(t_{1}^{\prime }\right)}∥\cdots ∥A_{d}^{\left(t_{d}^{\prime }\right)}]$ and $h^{\prime }=\sum_{i=1}^{k}{\left(-1\right)}^{\tau_{i}^{\prime }}m_{i}^{\prime }a_{i}$.

**Analysis**: We argue that from A’s perspective, it cannot distinguish between the experiment simulated by C and the actual scheme. The reasons are as follows:

(1) In the simulated game, not all matrices $A_{i}^{\left(b\right)}$ used in constructing the matrix $F_{t}$ are randomly sampled from $Z_{q}^{n\times m}$, some are generated by TrapGen(n,m,q). According to Theorem 1, matrices produced by this algorithm are statistically indistinguishable from the uniform distribution over $Z_{q}^{n\times m}$.
(2) In the simulated game, the vectors are computed as $a_{1}=F_{t}v_{1}\;\mathrm{mod}\;q,a_{2}=F_{t}v_{2}\;\mathrm{mod}\;q,\ldots ,a_{k}=F_{t}v_{k}\;\mathrm{mod}\;q,$ where the vectors $v_{1},v_{2},\ldots ,v_{k}$ are obtained by running $\mathrm{SampleDom}(1^{(d+1)m},\delta \sqrt{\frac{2}{k}})$. By Theorem 5, the vectors $a_{1},a_{2},\ldots ,a_{k}$ are statistically indistinguishable from vectors uniformly sampled from $Z_{q}^{n}$.
(3) In Case 1 of the signature query phase, the signature is generated in the same way as in the actual scheme.
(4) In Case 2 of the signature query phase, the simulated signatures $\sigma (\upsilon_{j}^{\left(i\right)})$ and $\sigma (\gamma_{j}^{\left(i\right)})$ are, by Lemmas 2 and 3, statistically close to samples drawn from distribution $D_{Z^{(d+1)m},\delta }$. Moreover, since $F_{t}\sigma \left(\upsilon_{j}^{\left(i\right)}\right)\;\mathrm{mod}\;q=F_{t}\sum_{u=1}^{k}{\left(-1\right)}^{\tau_{u}^{\left(i\right)}}\upsilon_{ju}^{\left(i\right)}v_{u}\;\mathrm{mod}\;q=\sum_{u=1}^{k}{\left(-1\right)}^{\tau_{u}^{\left(i\right)}}\upsilon_{ju}^{\left(i\right)}F_{t}v_{u}\;\mathrm{mod}\;q=\sum_{u=1}^{k}{\left(-1\right)}^{\tau_{u}^{\left(i\right)}}\upsilon_{ju}^{\left(i\right)}a_{u}=h\left(\upsilon_{j}^{\left(i\right)}\right)$ and $F_{t}\sigma \left(\gamma_{j}^{\left(i\right)}\right)\;\mathrm{mod}\;q=F_{t}\sum_{u=1}^{k}{\left(-1\right)}^{\tau_{u}^{\left(i\right)}}\gamma_{ju}^{\left(i\right)}v_{u}\;\mathrm{mod}\;q=\sum_{u=1}^{k}{\left(-1\right)}^{\tau_{u}^{\left(i\right)}}\gamma_{ju}^{\left(i\right)}F_{t}v_{u}\;\mathrm{mod}\;q=\sum_{u=1}^{k}{\left(-1\right)}^{\tau_{u}^{\left(i\right)}}\gamma_{ju}^{\left(i\right)}a_{u}=h\left(\gamma_{j}^{\left(i\right)}\right)$, it follows from Theorem 5 that $\sigma (\upsilon_{j}^{\left(i\right)})$ and $\sigma (\gamma_{j}^{\left(i\right)})$ are also approximately distributed as $D_{\Lambda_{q}^{h(\upsilon_{j}^{\left(i\right)})}\left(F_{t}\right),\delta }$ and $D_{\Lambda_{q}^{h(\gamma_{j}^{\left(i\right)})}\left(F_{t}\right),\delta }$, respectively.
(5) Verifying that $F_{t}\sigma_{j}^{\left(i\right)}\;\mathrm{mod}\;q=h_{j}^{\left(i\right)}$ holds, the process is as follows:
  $$\begin{array}{cc} F_{t}\sigma_{j}^{\left(i\right)}\;\mathrm{mod}\;q & =F_{t}(\sigma \left(\upsilon_{j}^{\left(i\right)}\right)+\sigma \left(\gamma_{j}^{\left(i\right)}\right)\;\mathrm{mod}\;q\\ \; & =F_{t}\left(\sum_{u=1}^{k}{\left(-1\right)}^{\tau_{u}^{\left(i\right)}}\upsilon_{ju}^{\left(i\right)}v_{u}\;\mathrm{mod}\;q+\sum_{u=1}^{k}{\left(-1\right)}^{\tau_{u}^{\left(i\right)}}\gamma_{ju}^{\left(i\right)}v_{u}\;\mathrm{mod}\;q\right)\\ \; & =\sum_{u=1}^{k}{\left(-1\right)}^{\tau_{u}^{\left(i\right)}}\left(\upsilon_{ju}^{\left(i\right)}+\gamma_{ju}^{\left(i\right)}\right)F_{t}v_{u}\;\mathrm{mod}\;q=\sum_{u=1}^{k}{\left(-1\right)}^{\tau_{u}^{\left(i\right)}}m_{ju}^{\left(i\right)}a_{u}=h_{j}^{\left(i\right)}. \end{array}$$
(6) Next, we verify that $∥\sigma_{j}^{\left(i\right)}∥<2L\delta \sqrt{2k(d+1)m}$ holds. By Lemma 1, the probability that $∥\sigma \left(\upsilon_{j}^{\left(i\right)}\right)∥=∥\sum_{u=1}^{k}{\left(-1\right)}^{\tau_{u}^{\left(i\right)}}\upsilon_{ju}^{\left(i\right)}v_{u}\;\mathrm{mod}\;q∥\leq \delta k\sqrt{\frac{2}{k}}\sqrt{(d+1)m}\leq L\delta \sqrt{2k(d+1)m}$ and $∥\sigma \left(\gamma_{j}^{\left(i\right)}\right)∥=∥\sum_{u=1}^{k}{\left(-1\right)}^{\tau_{u}^{\left(i\right)}}\gamma_{ju}^{\left(i\right)}v_{u}\;\mathrm{mod}\;q∥\leq \delta k\sqrt{\frac{2}{k}}\sqrt{(d+1)m}\leq L\delta \sqrt{2k(d+1)m}$ is overwhelming. Hence it follows that $∥\sigma_{j}^{\left(i\right)}∥=∥\sigma \left(\upsilon_{j}^{\left(i\right)}\right)+\sigma (\gamma_{j}^{\left(i\right)}∥\leq 2L\delta \sqrt{2k(d+1)m}$.

Now, let’s demonstrate how to obtain a solution for an instance of the ${\mathrm{SIS}}_{q,n,(2d+1)m,\beta }$ problem. Regardless of whether the forgery of A is of type 1 or type 2, we have

$$\begin{array}{cc} F_{t^{\prime }}\sigma^{\prime }\;\mathrm{mod}\;q & =h^{\prime }=\sum_{j=1}^{l}c_{j}h_{j}=\sum_{j=1}^{l}c_{j}\left(\sum_{u=1}^{k}{\left(-1\right)}^{\tau_{u}^{\prime }}m_{ju}^{\prime }a_{u}\right)\\ \; & =\sum_{u=1}^{k}{\left(-1\right)}^{\tau_{u}^{\prime }}\left(\sum_{j=1}^{l}c_{j}m_{ju}^{\prime }\right)a_{u}=\sum_{u=1}^{k}{\left(-1\right)}^{\tau_{u}^{\prime }}m_{u}^{\prime }a_{u}\\ \; & =F_{t^{\prime }}\sum_{u=1}^{k}{\left(-1\right)}^{\tau_{u}^{\prime }}m_{u}^{\prime }v_{u}\;\mathrm{mod}\;q=F_{t^{\prime }}v^{\prime }, \end{array}$$

where $v^{\prime }=\sum_{u=1}^{k}{\left(-1\right)}^{\tau_{u}^{\prime }}m_{u}^{\prime }v_{u}\;\mathrm{mod}\;q$. The fifth equality is due to the fact that $m^{\prime }=\left(m_{1}^{\prime },m_{2}^{\prime },\cdots ,m_{k}^{\prime }\right)=\sum_{j=1}^{l}c_{j}m_{j}^{\prime }$, which implies that $m_{u}^{\prime }=\sum_{j=1}^{l}c_{j}m_{ju}^{\prime }$. Then $F_{t^{\prime }}\left(\sigma^{\prime }-v^{\prime }\right)=0\;\mathrm{mod}\;q$. From [37], we know that $Pr\left[\sigma^{\prime }\neq v^{\prime }\right]\geq 1-2^{-\omega (\log n)}$, which means $\hat{v}=\sigma^{\prime }-v^{\prime }$ is a solution to the ${\mathrm{SIS}}_{q,n,(d+1)m,\beta }$ problem.

According to SampleDom, we have $∥v^{\prime }∥=∥\sum_{u=1}^{k}{\left(-1\right)}^{\tau_{u}^{\prime }}m_{u}^{\prime }v_{u}\;\mathrm{mod}\;q∥\leq 2L\delta \sqrt{2k(d+1)m}$, which implies $∥\sigma^{\prime }-v^{\prime }∥\leq ∥\sigma^{\prime }∥+∥v^{\prime }∥\leq 4L\delta \sqrt{2k(d+1)m}=\beta$.

Note that $F_{t^{\prime }}=[A_{0}∥A_{1}^{\left(t_{1}^{\prime }\right)}∥\cdots ∥A_{d}^{\left(t_{d}^{\prime }\right)}]=[A_{0}∥U_{1}^{\left(t_{1}^{\prime }\right)}∥\cdots ∥U_{d}^{\left(t_{d}^{\prime }\right)}]$, while in the instance we have $F=[A_{0}∥U_{1}^{\left(0\right)}∥U_{1}^{\left(1\right)}∥U_{2}^{\left(0\right)}∥U_{2}^{\left(1\right)}∥\ldots ∥U_{d}^{\left(0\right)}∥U_{d}^{\left(1\right)}]$. Therefore, we “embed” the missing $\left\{U_{i}^{1-t_{i}^{\prime }}\right\}$ into $F_{t^{\prime }}$ to form the target matrix *F*. To match the dimension of the vector v with *F*, we pad the corresponding positions in $\hat{v}$ (which correspond to the newly inserted matrix blocks) with zero vectors of appropriate dimensions. This yields Fv=0modq, and at this point we have $∥v∥\;=\;∥\hat{v}∥$.

Since the probability of the game not aborting is $\frac{1}{T}$, combining the above analysis gives the advantage of C in solving the ${\mathrm{SIS}}_{q,n,(2d+1)m,\beta }$ problem as $\frac{(1-2^{-\omega (\log n)})\cdot \varepsilon }{T}$. This advantage is non-negligible under the given parameters, which contradicts the hardness assumption of the SIS problem. Therefore, the proposed scheme satisfies forward-secure unforgeability. □

## 5. Comparative Analysis

To ensure the comparability of the schemes, we select the recent lattice-based LHS scheme [19] and the lattice-based FSLHS scheme [32], and analyze them together with our scheme in four aspects: security model, forward security, underlying assumptions, and efficiency. The detailed comparison results are summarized in Table 2.

As shown in Table 2, all three schemes rely on the hardness of SIS to ensure security. Among them, our scheme and the scheme in [19] are proven secure in the SM, while the scheme in [32] is proven secure in the ROM. However, the scheme in [19] does not satisfy forward security.

In terms of efficiency, as shown in Table 2, we compare the three schemes from three dimensions: public key size, signature size, and signing time. In terms of public key length, our scheme is equivalent to that in [32] and smaller than that in [19].

To provide an intuitive comparison of signature sizes, we take the 128-bit security level as an example, with parameters set as n=512, $q\approx 2^{32}$, binary tree depth d=10, and m=6nlogq. The specific values are calculated according to the signature length formula of each scheme, and the results are shown in Table 3.

As can be seen from Table 3, the signature size of our scheme is approximately *d* times that of the other two schemes. This additional overhead mainly stems from the binary tree forward security mechanism introduced to achieve provable security in the standard model.

Regarding the signing time, to mitigate the impact of different devices, we analyze the time complexity of the signing algorithms. For simplicity, we ignore the complexity of hash functions and multiplications, which have a minor impact on the signing algorithm. According to [38], the time complexity of the SamplePre algorithm is $T_{sp}=O\left(m^{2}\right)$. Let $T_{\mathrm{ours}}$, $T_{\mathrm{Guo}}$, and $T_{\mathrm{Wu}}$ denote the signing time complexities of our scheme, the scheme in [19], and the scheme in [32], respectively. Under the same security parameters, the relationship among the three is $T_{\mathrm{Wu}}<T_{\mathrm{Guo}}<T_{\mathrm{ours}}$. This indicates that our signing time is not only longer than that in [32], but also longer than that in [19] due to the binary tree mechanism.

In summary, compared with the scheme in [19], our scheme achieves a smaller public key size and is resilient to key leakage attacks. Meanwhile, our scheme provides stronger security guarantees than the scheme in [32], which is only proven secure in the ROM.

Based on the above comparative results, the next section will use the concrete parameters (e.g., n=512, $q\approx 2^{32}$, d=10) and performance estimates (signature size ≈ 480 MB) set in this section to further analyze the feasibility of our scheme in a smart grid distributed data acquisition system.

## 6. Application Feasibility Analysis: Smart Grid Data Acquisition

To comprehensively evaluate the practicality and applicability of the proposed scheme, this section first takes the smart grid data acquisition system as a concrete example and conducts a feasibility analysis based on the performance data from Section 5. Subsequently, we further discuss the potential applications of the scheme in other typical scenarios.

**Scenario description**: Consider a large-scale smart grid system consisting of a massive number of smart meters, multiple regional aggregation nodes, and a central data center. The workflow follows the five stages defined in our scheme: setup, key update, signing, combination, and verification. In each time period *t*, each smart meter signs its collected data vector $m_{i}$ using its current secret key $sk_{t}$ and sends $\left(m_{i},\sigma_{i}\right)$ to its regional aggregation node. Without accessing any private keys, the aggregation node combines *l* signatures (where l<L, and *L* is the maximum batch size) into an aggregated signature $\sigma =\sum c_{i}\sigma_{i}$ and aggregated data $m=\sum c_{i}m_{i}$. The data center then verifies the aggregated data packet (m,σ,τ) using the public key pk. A successful verification proves that the aggregated data originates from legitimate meters and has not been tampered with.

**Performance feasibility analysis**: Using the parameter settings from Section 5 (128-bit security level, n=512, $q\approx 2^{32}$, d=10, m=6nlogq), we evaluate the suitability of our scheme for the smart grid scenario.

Signature size: The signature size of our scheme is approximately 480 MB, which is *d* times larger than that of [19,32]. In the smart grid environment, each aggregation node processes up to *l* signatures per time period. After combination, the aggregated signature size remains the same as a single signature (480 MB), rather than being *l* times larger. Therefore, the transmission overhead from the aggregation node to the data center is a constant factor per batch, which is feasible in wired or high-bandwidth industrial networks.

Signing time: According to Table 2, when d=10, the signing time complexity of our scheme is approximately $2{\left(d+1\right)}^{2}T_{sp}=242T_{sp}$. In typical lattice-based implementations, $T_{sp}\approx 0.1$–1 s (depending on the specific implementation and hardware), so the signing time per smart meter ranges from 24.2 to 242 s. This is suitable for low-frequency reporting scenarios, such as smart meters transmitting data every 15–30 min. For high-frequency or real-time applications, this overhead would be prohibitive.

Key update: The key update operation is performed locally on each meter and does not involve communication, thus it does not affect network scalability.

Based on the above analysis, our scheme is suitable for smart grid deployment scenarios that satisfy the following conditions:

- **Low-frequency reporting:** The time interval between consecutive signatures should be at least several minutes to accommodate the signing time of 24.2–242 s.
- **Need for forward security in the standard model:** Our scheme is intended for applications where key leakage is a major security concern and security under the random oracle model is insufficient.
- **Sufficient bandwidth:** The 480 MB aggregated signature requires adequate network capacity between the aggregation nodes and the data center. This is typically available in industrial-grade smart grid backhaul networks.

In summary, our scheme enables regional aggregation nodes to compute valid linear combinations of signatures without knowing any private key, while ensuring that the data center can verify the integrity and authenticity of the aggregated data. Even if the private keys of some smart meters are compromised, previously signed data remain unforgeable due to forward security. The security of our scheme reduces to the hardness of the SIS problem on lattices, thus resisting quantum attacks. The quantitative analysis in this section shows that for low-frequency reporting scenarios, the signing time and signature size are practically feasible, making our scheme a viable option for secure and verifiable data aggregation in smart grids.

### Other Potential Application Scenarios

Beyond smart grid data acquisition, the proposed scheme can also be applied to scenarios such as industrial IoT data aggregation, distributed healthcare monitoring, vehicular network security communication, and blockchain cross-chain verification. These application scenarios share the common characteristic of requiring data aggregation and verification at the edge side, while also demanding forward security to cope with the risk of key leakage.

**Industrial IoT data aggregation**: In Industry 4.0 environments, multiple sensors periodically transmit production data to edge gateways. Our scheme enables edge gateways to perform linear combinations of multiple sensor signatures without accessing private keys, generating aggregated signatures for transmission to central monitoring systems. Even if certain sensor nodes’ private keys are compromised in the future, the integrity of historical data remains protected due to forward security.

**Distributed healthcare monitoring systems**: In remote healthcare scenarios, multiple wearable devices periodically upload patients’ physiological data to hospital data centers. Our scheme supports hospital edge servers in securely aggregating data from multiple devices for the same patient, while ensuring that even if device private keys are leaked, patients’ historical health records cannot be tampered with.

**Vehicular networks security communication**: In intelligent transportation systems, multiple vehicles periodically send location and status information to Roadside Units (RSUs). Our scheme can be used for RSUs to aggregate and verify data from multiple vehicles, ensuring the authenticity and integrity of traffic data while providing forward security against potential vehicle device compromises.

**Blockchain cross-chain data verification**: In multi-chain architectures, different blockchain networks need to verify each other’s data. Our scheme can be employed to build cross-chain data verification mechanisms, allowing verification nodes to perform linear combination verification of transaction data from multiple source chains, while ensuring that even if certain verification nodes’ private keys are compromised, historical verification records remain unforgeable.

**Applicability conditions**: It should be emphasized that due to the signature size of approximately 480 MB (at 128-bit security level), this scheme is primarily suitable for resource-rich edge gateway devices rather than ultra-low-power micro-sensors. These application scenarios typically share the following characteristics:

- Low-frequency data reporting
- Stable power supply
- Sufficient storage capacity (GB-level)
- High-bandwidth network connections

## 7. Conclusions

This paper investigates the FSLHS scheme to address the security challenges posed by key leakage. Its main contribution lies in constructing the first provably secure lattice-based FSLHS scheme in the SM, filling the gap in existing research that lacks security proofs in the SM. Through a comparative analysis with existing schemes in terms of security model, underlying assumptions, and efficiency, we demonstrate the security advantages of our scheme while also pointing out its efficiency limitations. To verify the practicality of the proposed scheme, we apply it to a smart grid data acquisition system, analyze its feasibility under specific performance metrics, and present several potential application scenarios. It should be noted that this scheme is not suitable for high-frequency metering or ultra-low-power devices; therefore, it is primarily designed for edge-gateway-level devices with sufficient computational resources and storage capacity. In future work, we plan to: conduct performance evaluation and energy consumption measurement on real high-end edge computing hardware platforms; further optimize the signature size to make it applicable to a wider range of IoT devices; and extend forward security to certificateless and identity-based LHS frameworks, thereby enriching the cryptographic primitives for building network data authentication infrastructure that resists key leakage risks. 

## Figures and Tables

**Table 1:** Symbols and meanings.

Symbol	Meaning
O(·)	f(n)=O(g(n)) means ∃c>0 and $n_{0}\in N$ such that for $\forall \;n\geq n_{0}$, f(n)≤c·g(n).
$\tilde{O}\left(\cdot \right)$	$f\left(n\right)=\tilde{O}\left(g\left(n\right)\right)$ means $f\left(n\right)=O(g\left(n\right)\cdot {\left(\log n\right)}^{c})$ for some constant *c*.
ω(·)	f(n)=ω(g(n)) means that for ∀c>0, $\exists \;n_{0}\in N$ such that f(n)>c·g(n) for $n\geq n_{0}$.
poly(n)	$f\left(n\right)=O\left(n^{c}\right)$ for some constant *c*.
negl(n)	$f\left(n\right)=O\left(n^{-c}\right)$ for c>0.
Pr[Event]≥1−negl(n)	The event occurs with overwhelming probability.
[k]	The set {1,2,3,…,k}.
Bold lowercase letters	Vectors. e.g., z, h.
uppercase letters	Matrices. e.g., *F*, *H*.
$h=\left(\begin{array}{c} h_{1}\\ \vdots \\ h_{n} \end{array}\right)$	An *n*-dimensional column vector.
∥h∥	The $l_{2}$-norm of a vector h.
$H=(h_{1},\ldots ,h_{m})$	An n×m matrix composed of column vectors $h_{i}$.
$\tilde{T}$	The Gram–Schmidt orthogonalization of a matrix *T*.
y←D	y is drawn uniformly at random according to the distribution D.
$y\overset{\$}{⟵}S$	y is derived from uniform sampling over the set S.
y←Alg(x)	Running algorithm Alg with input *x* yields output *y*.

**Table 2:** Comparison of schemes.

Schemes	[19]	[32]	Our Scheme
Public key size	nmlogq+knlogq	nmlogq	nmlogq
Signature size	mlogq	mlogq+n	dmlogq
Signing time	$2T_{sp}$	$T_{sp}$	$2{\left(d+1\right)}^{2}T_{sp}$
Forward secure	No	Yes	Yes
Security model	SM	ROM	SM
Assumption	SIS	SIS	SIS

*n* is the number of rows in the matrix. *m* is the number of columns in the matrix, where m≥6nlogq. q>2 is a prime number; *k*: polynomial in *n*; *d*: binary tree depth; $T_{sp}$: time of one execution of algorithm SamplePre.

**Table 3:** Comparison of signature sizes at 128-bit security level.

Scheme	Signature Size	Approximate Numerical Value	Actual Size (MB)
[19]	mlogq	512×6×512×32	≈48
[32]	mlogq+n	512×6×512×32+512	≈48
Our scheme	d·mlogq	10×512×6×512×32	≈480

## Data Availability

No new data were created or analyzed in this study.
